# Is the Technological Quality of Old Durum Wheat Cultivars Superior to That of Modern Ones When Exposed to Moderately High Temperatures during Grain Filling?

**DOI:** 10.3390/foods9060778

**Published:** 2020-06-11

**Authors:** Francesco Giunta, Simona Bassu, Marina Mefleh, Rosella Motzo

**Affiliations:** 1 Dipartimento di Agraria, Sez. Agronomia, Coltivazioni Erbacee e Genetica, University of Sassari, Via de Nicola 1, 07100 Sassari, Italy; giunta@uniss.it (F.G.); mmefleh@uniss.it (M.M.); 2 European Commission, Joint Research Centre (JRC), 21027 Ispra VA, Italy; simona.bassu@ec.europa.eu

**Keywords:** durum wheat, old cultivars, protein percentage, gluten index, alveographic indices, gliadin:glutenin

## Abstract

The growing interest in old durum wheat cultivars, due to enhanced consumer attention on healthy, traditional products and low-input agricultural systems, partly relies on their different quality characteristics compared to modern cultivars. Nine Italian durum wheat cultivars from different breeding periods were compared in two late-sown (January) field trials in order to subject their grain filling period to high temperatures similar to those expected in the future. Late sowing moved anthesis forward by about 10 days and increased the mean temperature during grain filling by 1.3 °C compared to that obtained when using the common sowing period of November–December. In these conditions, old cultivars were on average less productive than modern ones (2.36 vs. 3.54 tons ha^−1^, respectively), had a higher protein percentage (13.8% vs. 11.1%), a lower gluten index (24.3% vs. 56.3%), and a lower alveographic W (baking strength) (64 vs. 100 J 10^−4^). The differences were partly associated to variations in the gliadins:glutenins ratio. It depended on the genotype whether the grain and semolina protein percentage and gluten strength compensated one another in terms of alveographic indices to give the dough a strength similar to that of the modern cultivars in the range of moderately high temperatures, which resulted from delayed sowing. Further studies aimed at exploring the genetic variability of quality traits in the large genetic pool represented by the several Italian old and intermediate durum wheat cultivars still available are therefore advisable.

## 1. Introduction

The global production of durum wheat (*Triticum turgidum* subsp. *durum* Desf.) in 2016 amounted to about 40 million tons. The European Union is the world leader in the production of durum wheat, with 9.4 million tons (t) produced each year. Italy is the main European producer, generating about 4 million tons, i.e., 10% of world and 41% of European Union production (International Grains Commission, May 2017). Pasta is the main product, although about 24% of global durum wheat production, reaching 70–90% in some Middle East countries, is used for bread-making [1].

In Italy, durum wheat breeding has substantially increased grain yields [2,3], although the increase in yield implies higher inputs and a greater risk of environmental pollution than in the past [4]. The combination of increasing public awareness of environmental issues with the growing consumer attention to healthy, traditional products and organic food, has led to a rediscovery of old durum wheat cultivars over the last few decades. These are particularly suited to organic systems and to the low-input agricultural systems typical of marginal areas [5,6], and in Italy they are still grown on small acreages [7]. In these agricultural systems, old cultivars are traditionally used to produce several specialty breads, which can be sold at very high prices, thus assuring high incomes for the farmers. This means that the recognized outstanding genetic pool represented by old Italian durum wheat cultivars [8] is not merely a source of genes for breeding but also contains some cultivars which can be grown directly.

Old durum wheat cultivars also differ from modern ones in terms of quality, as expressed by protein percentage and gluten strength. Regardless of the nitrogen fertilization rate, old durum wheat varieties are generally characterized by higher protein percentages compared with modern ones, because of their lower grain yields [2,9]. However, they have not benefited from the breeding work aimed at improving the pasta-making quality [10,11,12] and are therefore characterized by a lower gluten strength compared with modern cultivars [9,13,14]. Gluten strength describes the ability of the proteins present in the grain to form a satisfactory network in terms of continuity and strength [15] and—given similar protein percentages—depends on the types of glutenin and gliadin proteins and their ratio. When pasta is the final product, strong gluten dough is less sticky and produces superior cooked textural characteristics. In the case of bread, strong gluten is the prerequisite for obtaining an extensive viscoelastic matrix with good physical and handling properties, i.e., high resistance to extension and moderate extensibility [15].

In addition to the genotype, environmental conditions, particularly drought and temperature, contribute to determining variations in wheat quality traits. The effects of temperature on wheat quality have been generally studied with reference to modern cultivars of bread wheat and may be positive or negative depending on the level and duration of heat stress. In both bread [16,17] and durum wheat cultivars, protein percentage generally increases under high temperatures because dry matter accumulation in the grains is affected more than nitrogen accumulation. Gluten strength may increase [18,19] or decrease [20,21,22,23] mainly as a consequence of the effects of temperature on the relative amounts of glutenins and gliadins and on the amount of large-sized SDS (Sodium Dodecyl Sulphate)-unextractable polymers [24].

Understanding the effects of temperature on wheat quality is crucial in view of the expected global rise in average temperatures. Global climate change projections suggest that, in southern Europe, the mean temperature for the 2021–2050 period will increase by 1.0–1.5 °C [25,26]. Given the differences in quality traits between old and modern cultivars and the sensitivity of all quality traits to temperature, we can hypothesize that high temperatures will differentially affect the technological quality of old and new cultivars. Moreover, a different response to temperature of the two groups of cultivars is expected because old durum wheat cultivars flower later than modern ones [20]. Therefore, their grain filling period is exposed to higher temperatures than that of modern cultivars when sown at the same time.

Nine Italian durum wheat cultivars from different breeding eras were compared in two field trials to evaluate the effect of late sowing on grain yield and grain and semolina quality of old and modern cultivars. Late sowing combined with irrigation allowed us to expose the grain filling period to higher temperatures than those occurring with normal sowing dates, without the confounding effect of drought stress. 

## 2. Materials and Methods 

The study was carried out in 2005/06 and 2006/07 at the University of Sassari ‘Ottava’ experimental station (41°N; 8°E; 80 m above sea level). The environment is typically Mediterranean, with a long-term mean annual rainfall of 553 mm, mainly concentrated between October and April. The soil is sandy-clay-loam with a depth of about 0.6 m due to underlying layers of limestone (typic Xerochrepts).

The treatments consisted of nine Italian durum wheat landraces/cultivars from different breeding eras. Their names, geographic or genetic origins, and years of release are shown in Table 1. The four oldest constitutions (‘old’ group) are tall and belong to the ‘*mediterraneum*’ type [27]. Saragolla, Trigu murru, and Triminia were amongst the most cultivated landraces respectively in Apulia, Sardinia, and Sicily between 1800 and 1920. Then, the cultivar Senatore Cappelli, a pure line extracted from the North African population Jean Retifah, rapidly became the most important durum wheat in Italy, where it was cultivated on 60% of the total durum wheat-growing area until 1950 [28].

Its importance lead to its extensive use in the subsequent crosses, and in 1987 about 80% of the Italian durum wheat cultivars had Senatore Cappelli in their pedigree [29] included the two intermediate cultivars Ichnusa and Trinakrian cultivars are characterized by reduced height and earlier flowering in comparison with the oldest types, achieved by introgression from the ‘*syriacum*’ types. The three earliest constitutions, Creso, Svevo, and Claudio, are *Rht1* semi-dwarf cultivars (‘modern’ group). Creso was one of the first semi-dwarf cultivars released in Italy. It was widely grown for at least 30 years despite its lateness. The distinguishing trait of cultivar Svevo is its reputation for producing good quality pasta [13].

High Molecular Weight Glutenin Subunits (HMW-GS) were separated by SDS-PAGE (Sodium Dodecyl Sulphate-PolyAcrylamide Gel Electrophoresis), and protein extraction was done from 20 mg of durum semolina [30]. The identification of HMW-GS alleles was based on the classification proposed by Payne and Lawrence [31]. The cultivars compared were genetically homogeneous in terms of Low Molecular Weight Glutenin subunits (LMW-GS) pattern, as all shared the LMW-2 type, a genotype associated with strong gluten and superior pasta texture [32]. On the other hand, they were heterogeneous at the HMW-GS locus because the breeding work carried out in Italy substituted the HMW-20 and HMW-7 genotypes characteristic of the old and intermediate cultivars, with the 6 + 8 and 7 + 8 HMW genotypes.

Cultivars were compared in a randomized complete block design with three replications.

The sowing bed was prepared by ploughing to a depth of 0.25 m, followed by surface cultivation. In both seasons, the materials were sown (on 13 January in the season 2005/06 and on 5 January in the season 2006/07) with an 8-row planter at a density of 200 viable seeds m^−2^. Each 10 m^2^ plot consisted of eight 8.3 m rows, separated from one another by 0.15 m. Nitrogenous (80 kg N ha^−1^) and phosphoric fertilizer (92 kg P_2_O_5_ ha^−1^) were applied before sowing in the form of urea and ammonium bi-phosphate, and the plots were sprinkler-irrigated to prevent the development of any moisture stress. Weeds, pests, and diseases were chemically controlled. During the course of the experiment, weather conditions (rainfall, solar radiation, temperature) were recorded at a meteorological station located in an adjacent field.

Anthesis date was recorded as the time at which 50% of the spikes in a plot had visible anthers. Physiological maturity was set as stage 90 in the Zadoks’ Scale [33]. Plant height was defined as the distance from the ground to the tip of the spike (awns excluded) and was assessed at pre-harvest for four randomly chosen plants per plot. At maturity, each plot was sampled by cutting, at ground level, samples of 0.3 m^2^ (4.0 m × 0.15 m inter-row) from an inner row within the plot. The total biomass was obtained by weighing these air-dried samples. Spikes were then separated, counted and threshed, and the Harvest Index was calculated by determining the ratio between the grain and the total biomass weight. The number of spikes per m^2^ was obtained by dividing the number of spikes by the harvested area. Grain yield was calculated on a per plot basis.

Thousand-grain weight, protein percentage at 13% moisture content, test weight (hectolitre weight), and percentage of yellow berry were determined on mature grains. Samples of 50 kg per cultivar were milled with a traditional stone mill, following standard procedures for durum wheat. Semolina and flour quality were assessed by measuring nitrogen and protein percentage, gluten index (GI), alveographic indices, and gliadin-to-glutenin ratio (gli:glu). Nitrogen content was determined with the Kjeldahl method using a conversion factor of 5.7 to calculate the protein percentage [34]. The Chopin Alveograph was used to obtain gluten strength value (W) and the tenacity/extensibility ratio (P/L) from 250 g samples. Dough was formed by the addition of sufficient saline to give a final concentration of 2.5% *w/v* dry semolina/flour. Mixing times differed from those usually used for bread wheat. Wet and dry gluten contents and the GI were determined using the AACC Method 38–12 [34]. Gluten was separated from whole wheat meal by washing (Glutomatic 2200, Perten Instruments AB, Huddinge, Sweden) and then centrifuged at 6k rpm to force the wet gluten through a specially constructed sieve under standard conditions. The total weight of this gluten was taken as the gluten quantity. The percentage of gluten unable to pass through the sieve after centrifugation was taken as GI. In very weak gluten samples, all substances pass through the sieve (GI = 0); when nothing passes through, GI = 100. GI correlates well with the manual test commonly used in the Italian pasta industry [35]. Dry gluten percentage was obtained by reweighing the gluten after drying the wet gluten for a few minutes. Semolina/flour proteins from 2 g samples were then separated into four classes: albumins, globulins, gliadins, and glutenins, using standard methods [36]. The representation of each class was expressed as a percentage of the total semolina/flour protein, and the gli:glu was calculated from the sample’s gliadin and glutenin content.

The significance of the differences between the years, the three groups of cultivars, and their interaction were analyzed using the cultivar means as replications. Least squared means were presented to correct for the unequal number of cultivars in the three groups. Where the F test suggested a significant treatment effect, means were separated by the Student’s t-test (*p* < 0.05). Overall differences in yield and quality traits of cultivar groups were investigated by principal component analysis (PCA) executed on the correlation matrix of the cultivar traits, followed by a rotation of components (Robust Principal Component Analysis, RPCA). Component loadings were calculated as simple correlations (using Pearson’s *r*) between the components (i.e., component scores for each cultivar) and the original variables [37]. Statistical analysis was performed using the R Package [38].

## 3. Results

Due to the general lack of genotype × environment interaction, only main effects are reported in the tables.

### 3.1. Temperature and Phenological Traits

In the two experimental years, differences in crop growth and development were mainly driven by temperature, as water and nutrients were provided in order to avoid any water or nutritional stress. A slightly higher mean temperature was recorded between sowing and maturity in the 2007 growing season compared with the 2006 season, with average mean temperatures of 14.9 °C vs. 13.8 °C, respectively (Table 2). The mean temperature during grain filling was also higher in 2007, and the difference in this case was attributable to the notably higher minimum temperature (14.3 °C vs. 12.9 °C). The lower temperatures of 2006 were likely the cause of the three day longer grain filling period recorded that year. Surprisingly, peak maximum temperatures in this same period were higher in 2006, peaking at 39 °C on some occasions.

In both seasons, old cultivars were later than intermediate and modern cultivars both in anthesis and maturity, such that a negative relationship was calculated between these pheno-stages and the year of release (*r* = −0.73* for anthesis and *r* = −0.70* for maturity). These differences were mirrored in an almost similar duration of grain filling in the three groups.

The later anthesis of old cultivars moved their grain filling period forward; with the result of subjecting this stage of growth to temperatures that were 0.9 °C higher in 2006 and 0.6 °C higher in 2007 compared with those observed for the modern cultivars. Consequently, a negative relationship was calculated between the year of release and the temperatures recorded during grain filling.

In the last days of grain filling for the older cultivars, up to 10 days of temperatures above 30 °C, at least in 2006, were recorded.

### 3.2. Grain Yield and Grain Quality

A greater grain yield was obtained in 2006 as a result of both a greater biomass and a greater harvest index (Table 3), likely due to the fact that the soil of the field sown in this year was deeper and more fertile. Grain weight was also higher in 2006 (by 6 mg), as a consequence of the 3 days-longer grain filling duration.

These larger grains probably contributed to a lower protein percentage via a dilution effect, whereas grain quality was better in this year in terms of both test weight and yellow-berry incidence. The very low mean grain weight recorded in 2007 (below 40 mg) was indicative of shrunken grains, as pointed out by the very low absolute weight (lower than 80 kg hl^−1^).

The positive effect of breeding on grain yield via the increased harvest index at similar levels of above-ground biomass was demonstrated by the corresponding correlations with the year of release. The increase in grain yield with the year of release was in turn accompanied by a significant increase in grain number m^−2^ but similar grain weight. Intermediate cultivars, on the contrary, were more productive than old cultivars because of their higher grain weight at similar levels of grain number m^−2^. Grain protein percentage was also clearly affected by breeding, as shown by its progressive decrease from the old to the intermediate and the modern group and also by its negative correlation with the year of release. The superior quality of old cultivars in comparison with modern ones was also clear when expressed in mg of N per grain, with 0.75 mg N grain^−1^ in the modern cultivars and 0.95 mg N grain^−1^ in the old ones. The reason for the lower protein percentage of the intermediate cultivars compared to the old ones can be found in the dilution effect of their larger grains with the same mg of nitrogen per grain. The increase in grain yield with the year of release was one of the reasons for the decrease in protein percentage, as a negative relationship was calculated between these two traits (*r* = −0.84**, *n* = 9). The cultivar rank in protein percentage was very consistent between years, despite the difference in environmental conditions, as shown by a correlation coefficient of 0.75* between the protein percentages of the nine cultivars in 2006 and in 2007.

### 3.3. Semolina Quality

Raw material was ground by a stone mill and sifted into a main product, semolina, and a by-product, referred to as ‘flour’, since it appears as white and fine as bread wheat flour (Table 4). Semolina yield value in 2007 was very low, both absolutely and when compared with the value obtained in 2006 and counterbalanced by a corresponding 27% increase in bran yield.

The lack of interaction for the quality traits shown in Table 5 means that all the cultivars, irrespective of the year of release, reacted in a similar way to the different thermal conditions characterizing the grain filling period of the two seasons. The season effect on grain protein percentage (Table 3) was mirrored in semolina protein content, which was consistently lower in 2006 than in 2007 (Table 5). It is interesting to note that the higher protein percentage of 2007 was associated with an increase in the albumin-globulin fraction from 35% to 43% of the total proteins, whereas gliadin percentage did not vary and glutenin percentage decreased from 35% to 37%.

By contrast, gluten strength, as measured by GI, was higher in 2006 than in 2007; this was probably a consequence of the higher glutenin percentage obtained in 2006 which decreased, although not significantly, the gli:glu ratio. No other quality trait was affected by the season. 

Protein quantity and quality were affected by breeding in opposite directions, as the decrease in semolina protein percentage going from the old to the modern cultivars was counterbalanced by a corresponding increase in gluten strength, either expressed as GI or W. No differences between groups of cultivars were detected in the percentage of the different protein fractions and in the other alveographic indices, despite the strong positive relationship between W and P, the alveographic index associated to dough tenacity (*r* = 0.91**, *n* = 9).

### 3.4. Phenology, Grain Yield, Grain Quality, and Semolina Quality

**Figure 1:**
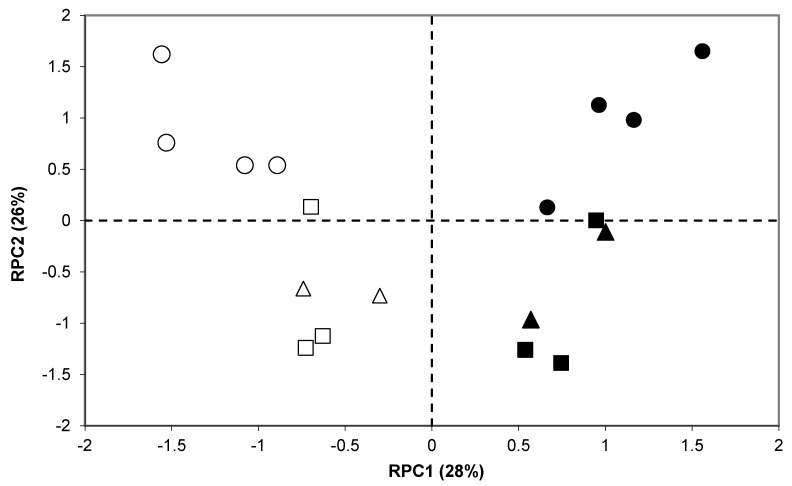
Principal Component Analysis graph for phenological, productive, and semolina quality traits; empty symbols: 2007; solid symbols: 2006; circles: old cultivars; triangles: intermediate cultivars; squares: modern cultivars.

When all productive and quality variables measured in the two seasons were subjected to a PCA analysis, a clear discrimination was observed between seasons and between old and modern cultivars (Figure 1). The difference between the two seasons was captured by the first component (RPC1) and comprised variations induced in grain yield, grain filling duration, grain weight, absolute weight, semolina yield, and semolina protein percentage (Table 6).

RPC2 explained about the same proportion of variance as RPC1, but captured differences between cultivars, which resulted from breeding and which were linked to their anthesis date and its effects on the thermal conditions during grain filling. As it was associated with semolina protein percentage, GI, alveographic L and gli:glu, this same axis establishes a link between phenology and these quality traits.

## 4. Discussion

Sowing in January moved anthesis of modern cultivars forward by about 10 days with respect to the anthesis date of 24 April, calculated as the mean value of 25 modern cultivars grown for nine years in this same environment and under the common sowing date of November–December [39]. Based on the long-term meteorological data set from Ottava for the 1961–1990 period, the mean temperature over the 45 days following anthesis, with the April 24 as the anthesis date, is 16.8 °C; however, if the anthesis is moved to May 4, it rises to 18.1 °C. The increase of 1.3 °C obtained by delaying sowing date was therefore within the 1.0–1.5 °C increase predicted for the 2021–2050 period for the Mediterranean countries [27,28]. Of course, temperature was not the only environmental factor affected by the delayed sowing because the photoperiod and rainfall pattern also changed, but irrigation prevented the confounding effect of a drought stress to be expressed.

Any quality evaluation of wheat begins with the raw material, i.e., the grain, as grain composition in terms of protein content, composition, and aggregation level, influences the dough characteristics and the quality of the final product. Grain protein percentage contributes the most (40%) to the EU Quality index for durum wheat (European Commission Regulation No. 2237/2003, 23 December 2003), followed by gluten strength (30%). In some countries, grain protein content influences the amount of money paid to wheat farmers due to the importance of this trait for the quality of both pasta and bread. Grain protein percentage can vary as a consequence of genotype, environment, or genotype × environment and, as pointed out in most of the papers estimating the contribution of these parameters to protein percentage variation in sets of modern cultivars, the environment is the main component [40,41]. On the other hand, genotypic variation for protein percentage is usually high when old and modern cultivars are compared, as observed in this experiment and others [2,3,9,42]. This is due to the constantly lower protein percentage of modern cultivars under varying environmental conditions, such as the high temperatures obtained in this experiment. The decrease in grain protein percentage observed in modern Italian durum wheat cultivars compared with older constitutions cannot be analyzed, as it is often done, without any reference to the corresponding variation in productivity, as the two traits are generally negatively associated [2,43]. Hence, the negative association between protein percentage and year of release of the cultivars should be simply considered as a consequence of the improvement in grain yield of durum wheat brought about by the introgression of *Rht* genes and the consequent increase in harvest index and in grain number m^−2^, as already discussed by Giunta et al. [2]. In the present experiment, the decrease in protein percentage was mediated by the increase in grain number m^−2^, because the higher grain number m^−2^ of modern constitutions was mirrored in less mg of nitrogen per grain, which in the end resulted in a higher protein percentage, since grain weight was not affected by breeding, as already discussed by Motzo et al. [44], at least as a consequence of the introgression of the *Rht* genes. Interestingly, the intermediate group of cultivars showed a lower protein percentage than the old cultivars, deriving from their larger grains at similar levels of grain number per m^−2^ and thus of mg of nitrogen per grain. The strong link between grain number m^−2^, harvest index, and grain protein percentage, also supported by the PCA analysis, thus explains the consistent cultivar ranking observed in grain protein percentage between the two seasons, indicative of the importance of the genetic control of this trait, although mediated by the genetic improvement in grain yield, also under high temperatures during grain filling.

Interestingly, while the genotypic differences in protein percentage were attributable to the sole storage proteins, i.e., the gluten fraction, the higher protein percentage observed in 2007 compared to the preceding season was due to a higher fraction of metabolic proteins, i.e., globulins and albumins, with an unchanging level of gluten percentage but a decrease in the glutenin fraction. As structural/metabolic protein fractions accumulate mainly during the early phase of grain growth, i.e., from anthesis to approximately 20 days after anthesis when most endosperm cells are still dividing, whereas storage-protein fractions accumulate later, from about 6 days after anthesis to the end of grain filling [45,46], we can hypothesize that the shorter grain filling duration deriving from the higher temperatures of 2007 impacted the deposition of storage proteins, glutenin in particular, more than metabolic proteins.

In the present study, gluten strength, which is the second most important trait in defining wheat quality, was evaluated using both the GI method [35] and the alveographic parameters. GI is an index developed for durum wheat and is generally used to evaluate its pasta-making quality, while the second method was originally developed for bread wheat to evaluate its bread-making quality. Either measured as GI or alveographic W, gluten was stronger in modern cultivars than in old ones as a consequence of both their genotypic differences at the *Glu B1* locus, and the differences in the environmental conditions to which their grain filling was exposed as a consequence of their different phenology.

In Italy, the improvement in the pasta-making quality of durum wheat through breeding has already been documented [3,9,13] and has implied a change in the glutenin composition, as glutenins are the key determinant for gluten strength [47,48]. Evidence for this change is provided by the sets of cultivars compared in this experiment, with most old and intermediate cultivars sharing the HMW-GS 20 genotype at the *Glu B1* locus, Creso, the oldest among modern cultivars, carrying the HMW-GS 6 + 8 genotype, and the other two modern cultivars possessing the HMW-GS 7 + 8 genotype. The 6 + 8 genotype has been identified as the best one for bread making by Ammar et al. [49]; by contrast, Pena et al. [12] and Boggini and Pogna [50] found the 7 + 8 genotype to be the best one for bread-making, in disagreement with Mefleh et al. [51], who showed that the 7 + 8 genotype is responsible for tough and dense bread.

For this reason, in our experiment, genotypic variation in GI was large, and mainly associated to the year of release of the cultivars and to the different kinds of glutenin molecules encoded by the different genes, in agreement with previous findings, [9,13,51]. According to Oikonomou et al. [52] both modern and intermediate cultivars can be classified in the medium range of GI (30–60%), despite the HMW-GS 20 genotype at the *Glu B1* locus distinguishing the intermediate cultivars. This observation highlights that no single allele is absolutely necessary for adequate gluten strength, which is the consequence of specific combinations of alleles [53], and that intermediate cultivars can be a source of potentially useful genetic variability to improve grain quality.

Our data did not show any difference between the cultivar groups in the proportion of the different protein fractions, although De Santis et al. [13] attributed the historical increase in GI of the Italian germplasm, not only to the introgression of the more favorable 6 + 8 and 7 + 8 alleles at the *Glu B1* locus, but also to a higher gli:glu ratio deriving from a greater expression of the B-type LMW-GS in terms of percentage of total storage proteins. On the other hand, the PCA analysis highlighted that the lateness of old cultivars exposed their grain filling to higher temperatures compared to the modern constitutions, and that this situation negatively affected GI by altering the gli:glu ratio via a decrease in glutenin percentage. Ultimately, therefore, our results agree with De Santis et al. [13]. In the light of our results, this greater level of expression can be attributed to the different environmental conditions occurring during grain filling, which were experienced by the two groups of cultivars because of their different phenology. Unfortunately, De Santis et al. [13] did not calculate the temperature characterizing the grain filling period of the two groups.

The observed environmental variation in GI, contradicting Sekuralak et al. [54] who did not find any relationship between GI and environmental factors, derived uniquely from the variation in gli:glu because the lower GI obtained in 2007 was associated to a lower glutenin percentage at the same level of gliadins, which changed the gli:glu ratio. The association between GI and gli:glu in durum wheat was already discussed by Fois et al. [14] and can be attributed to the asynchronous accumulation, not only of metabolic and storage proteins, but also of gliadins and glutenins. The earlier accumulation of gliadins compared to glutenins [55,56,57] means that any shortening of the grain filling period is expected to affect the balance between protein fractions [58]. Consequently, Fois et al. [14] found a linear decrease in the gli:glu ratio from anthesis to maturity. This means that any environmental stress causing a shortening of the grain filling period, such as the higher temperatures of 2007 or the future higher temperatures, could result in a decrease in gli:glu and, consequently, in GI.

Initially proposed for determining bread wheat dough quality, the alveograph has also become widely accepted internationally as an indicator of the gluten strength of durum wheat, in part because of the weak discrimination power of GI for moderate to low strengths of gluten [40]. Alveograms of durum wheat generally indicate high tenacity (P) relative to elasticity (L), with P/L ratios above 1.5 [15]. The P/L values obtained in this experiment were always less than 1.5 and within the range described by Sarpistein et al. [59] for a high loaf volume and a soft crumb. Our three groups of cultivars shared the same P, L, and P/L, contradicting Gallo et al. [60] who showed that P and L are higher for modern durum varieties compared with old varieties. The late sowing date of our study could be the reason for this discrepancy in the results.

The values of W recorded in this experiment (56–117 J 10^−4^) were similar to those reported by De Vita et al. [3] and Motzo et al. [61], but lower than those found in durum wheat by other authors [38,47,57]. According to Sissons [15], the old cultivars used in this experiment can therefore be classified as weak, and the intermediate and modern cultivars as weak to moderate in terms of gluten strength, as measured with W. Again, the different genotype at the *Glu B1* locus for modern and intermediate cultivars did not result in a different gluten strength.

**Figure 2:**
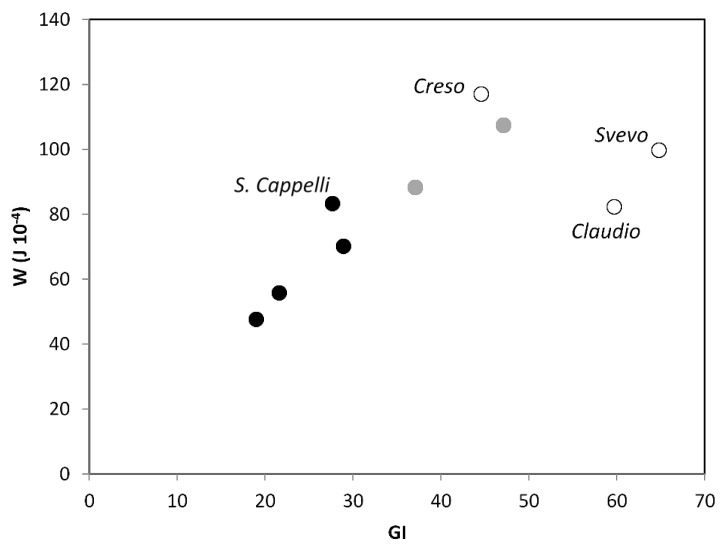
Relationship between gluten index (GI) and the alveographic index for gluten strength W.

The alveographic index W did not vary between seasons, contradicting Guzman et al. [22] and Fois et al. [14]. This lower sensitivity of W, compared with that of GI, to the higher temperatures characterizing the grain filling period of 2007 can be attributed to the fact that the alveographic indices evaluate the performance of the whole dough (gluten, plus non gluten proteins, plus starch), whereas GI is determined on a cleaned gluten matrix from which starch and non-gluten proteins are washed away. This means that the higher protein percentage counterbalanced the lower quality in terms of gli:glu and GI deriving from the higher 2007 temperatures. For these same reasons, the relationship between the cultivar mean values of W and those of GI was not perfectly linear (Figure 2) and the two modern cultivars, Claudio and Svevo, characterized by the lowest protein percentages, exhibited a W lower than expected in consideration of their high GI. Interestingly, the old cultivar Senatore Cappelli showed the same W as the modern cultivar Claudio because its lower GI (28 vs. 60) was counterbalanced by its higher protein percentage (14% vs. 11%). 

On the other hand, when the old cultivars grown in this experiment were compared as a group with the group of modern cultivars, dough strength as expressed by W was significantly higher in the modern cultivars in spite of their lower protein percentage. Given the large and outstanding genetic variability recognized by Royo et al. [8] in the Italian durum wheat pool—old cultivars included —compared with the dominating CIMMYT-derived germplasm, exploring this pool may present a way to find new and favorable genotypic combinations for quality traits.

## 5. Conclusions

In the range of moderately high temperatures explored, achieved through the use of delayed sowing with the aim of mimicking future increases in temperature, the technological quality of old Italian cultivars was higher compared with the modern cultivars in terms of grain and semolina protein percentage, but lower in terms of gluten strength. Whether the two components of wheat quality compensated one another in terms of alveographic indices to give dough of strength similar to that of the modern cultivars depended on the genotype. The intermediate group of cultivars, characterized by a grain quality and gluten strength comparable to modern cultivars, deserves further studies aimed at exploring their genetic variability for quality traits.

## Figures and Tables

**Table 1:** Origin, period of release, and gluten subunits of the durum wheat cultivars analyzed.

Cultivar	Geographic or Genetic Origin	Year of Release	HMW-GS *(Glu-B1)	LMW-GS **(Glu-B3)
Old group				
Saragolla	Apulia	1910	7	2
Trigu murru	Sardinia	1910	20	2
Triminia	Sicily	1920	7	2
Senatore Cappelli	Nord-african landrace Jean Retifah	1920	20	2
Intermediate Group				
Ichnusa	Biancale × Capeiti 8	1968	20	2
Trinakria	B14 × Capeiti 8	1970	20	2
Modern Group (semi-dwarf)				
Creso	Cpb144 × [(Yt54-N10-B)Cp2 63 Te3]	1974	6 + 8	2
Svevo	Sel. CIMMYT × Zenit sib	1996	7 + 8	2
Claudio	(Sel. Cimmyt × Durango) × (IS193B × Grazia)	1998	7 + 8	2

* HMW-GS, High Molecular Weight Glutenin Subunits; ** LMW-GS, Low Molecular Weight Glutenin Subunits.

**Table 2:** Phenological traits and temperatures during grain filling. ANOVA results, means, and coefficient of correlation (*r*) with the year of release.

ANOVA YEARS CULTIVARS	Anthesis	Maturity	Grain Filling Duration	Maximum Temperature	Minimum Temperature	Mean Temperature	Absolute Maximum Temperature	Days with Maximum Temperature >30 °C
	(doy) ^1^	(doy)	(days)	(°C)	(°C)	(°C)	(°C)	(n°)
Year	ns	*	*	ns	***	**	***	ns
Group	**	**	ns	**	**	**	*	**
Year × Group	ns	ns	ns	ns	ns	ns	ns	ns
2006	124	167	43	25.1	12.9	19.0	35.9	5.3
2007	123	164	40	25.0	14.3	19.7	33.0	3.5
Old	128 a	169 a	41	25.8 a	14.0	19.9 a	35.7	6.4 a
Saragolla	127	169	42	25.9	14.1	20.0	36.1	7.0
Trigu murru	127	166	39	25.2	13.6	19.4	33.9	4.0
Cappelli	127	169	42	25.9	14.1	20.0	36.1	7.0
Triminia	131	172	41	26.4	14.4	20.4	36.7	8.0
Intermediate	119 b	163 b	44	24.4 b	13.2	18.8 b	33.9	3.8 b
Ichnusa	119	163	44	24.4	13.2	18.8	33.9	3.0
Trinakria	120	163	44	24.5	13.3	18.9	33.9	3.0
Modern	123 b	165 b	42	24.9 b	13.5	19.2 b	33.9	3.0 b
Creso	127	166	40	25.4	13.8	19.6	33.9	5.0
Svevo	120	163	44	24.5	13.2	18.9	33.9	3.0
Claudio	123	166	43	24.9	13.6	19.2	33.9	4.0
Correlation with year of release (*r*)	0.73 *	0.70 *	ns	−0.74 *	−0.70 *	−0.73 *	−0.71 *	−0.67

^1^, day of the year; cultivar group means sharing the same letter do not differ significantly from one another (LSD Test at *p* ≤ 0.05); ***, significant at *p* ≤ 0.001; **, significant at *p* ≤ 0.01; *, significant at *p* ≤ 0.05; ns, not significant.

**Table 3:** Grain yield and grain quality. ANOVA results, means, and coefficient of correlation (*r*) with the year of release.

Cultivar/Year	Grain Yield	Above Ground Biomass	Harvest Index	Grain Weight	Grain Number	Grain Protein	Test Weight	Yellow Berry
	(t ha^−1^)	(t ha^−1^)		(mg)	(n° m^−2^)	(%)	(kg hl^−1^)	(%)
Year	***	***	***	***	***	***	***	***
Group	***	*	***	***	***	***	***	***
Year × Group	ns	ns	ns	ns	ns	ns	ns	ns
2006	3.71	11.1	0.34	44.3	8520	12.0	81.5	4.7
2007	2.58	9.4	0.28	38.4	6903	13.0	76.8	10.9
Old	2.36 c	10.3 a	0.23 c	39.0 b	6195 b	13.8 a	77.1 b	7.3 b
Saragolla	2.30	10.3	0.22	43.4	5357	12.9	78.4	6.2
Trigu murru	2.19	9.6	0.23	39.2	5639	14.6	74.8	5.8
Cappelli	2.43	10.7	0.21	41.4	5933	15.1	76.2	4.2
Triminia	2.51	10.5	0.25	32.1	7851	12.6	78.9	12.8
Intermediate	3.03 b	9.1 b	0.32 b	44.8 a	6757 b	12.0 b	79.3 a	6.4 b
Ichnusa	3.29	9.4	0.33	45.9	7195	11.4	80.8	5.5
Trinakria	2.76	8.9	0.30	43.6	6319	12.6	77.8	7.3
Modern	3.54 a	9.8 ab	0.37 a	38.4 b	9332 a	11.1 c	78.1 ab	14.3 a
Creso	3.48	10.1	0.35	41.8	8366	12.1	78.6	5.7
Svevo	3.58	10.2	0.37	33.6	10651	11.3	75.4	10.8
Claudio	3.57	9.1	0.39	40.0	8978	10.0	80.2	26.4
Correlation with year of release (*r*)	0.95 ***	ns	0.97 ***	ns	0.79 *	−0.82 **	ns	ns

Cultivar group means sharing the same letter do not differ significantly from one another (LSD Test at *p* ≤ 0.05); ***, significant at *p* ≤ 0.001; **, significant at *p* ≤ 0.01; *, significant at *p* ≤ 0.05; ns, not significant.

**Table 4:** Semolina yield.

	2006			2007		
Cultivar	Semolina	‘Flour’	Bran	Semolina	‘Flour’	Bran
	(%)	(%)	(%)	(%)	(%)	(%)
Saragolla	55.7	24.3	18.5	32.3	17.8	53.0
Trigu murru	61.1	23.9	18.8	39.6	16.7	45.2
Cappelli	57.1	24.6	19.6	29.2	19.3	39.6
Triminia	62.2	29.5	12.1	38.6	31.2	26.9
Ichnusa	58.4	23.9	20.4	41.9	18.0	49.7
Trinakria	57.8	31.6	9.6	38.9	19.6	43.2
Creso	55.8	28.1	10.8	41.1	16.8	43.7
Svevo	58.8	23.2	14.6	37.2	22.0	39.4
Claudio	57.4	25.1	17.1	38.1	14.9	46.2
Mean	58.3	26.0	15.7	37.4	19.6	43.0

**Table 5:** Semolina protein, gluten index, gli:glu and alveographic indices. ANOVA results, means, and coefficient of correlation (*r*) with the year of release.

Cultivar/Year	Protein	Gluten Index	Albumin Globulin	Gliadins	Glutenins	Gli:glu	P	L	W	P/L
	(% Dry Matter)		(%)	(%)	(%)		(mm H_2_O)	(mm)	(J 10^−4^)	
Year	***	**	***	ns	**	ns	ns	ns	ns	ns
Group	***	**	ns	ns	ns	ns	ns	ns	*	ns
Year × Group	ns	ns	ns	ns	ns	ns	ns	ns	ns	ns
2006	11.8	50.3	35.3	19.7	45.1	0.45	52	63	86	0.84
2007	13.2	31.5	43.0	20.3	36.7	0.56	55	64	89	0.96
Old	13.8 a	24.3 c	39.6	21.0	39.3	0.56	44	61	64 b	0.83
Saragolla	13.6	28.9	39.1	18.3	42.6	0.45	44	67	70	0.69
Trigu murru	13.8	21.6	39.5	17.9	42.6	0.44	43	46	56	0.99
Cappelli	13.9	27.6	36.7	25.4	37.9	0.67	50	78	83	0.62
Triminia	13.8	19.0	43.5	22.2	34.3	0.68	39	49	48	0.99
Intermediate	12.4 b	42.1 b	38.8	20.2	41.3	0.50	59	64	98 a	0.95
Ichnusa	11.7	37.0	37.2	23.3	39.5	0.60	59	59	88	1.03
Trinakria	13.0	47.1	40.3	16.6	43.1	0.40	60	69	108	0.87
Modern	11.3 c	56.3 a	38.9	18.9	42.2	0.46	57	66	100 a	0.92
Creso	11.5	44.6	40.6	17.8	41.7	0.46	72	56	117	1.30
Svevo	11.2	64.8	37.7	16.4	45.9	0.36	56	68	100	0.87
Claudio	11.1	59.7	38.5	22.6	39.0	0.58	43	75	82	0.58
Correlation with year of release (*r*)	−0.93 **	0.94 **	ns	ns	ns	ns	ns	ns	0.71 *	ns

Cultivar group means sharing the same letter do not differ significantly from one another (LSD Test at *p* ≤ 0.05); ***, significant at *p* ≤ 0.001; **, significant at *p* ≤ 0.01; *, significant at *p* ≤ 0.05; ns, not significant.

**Table 6:** Component loadings indicating the correlation between the components and the original variables.

Trait	RPC1	RPC2
Anthesis	ns	0.82 ***
Grain filling duration	0.64 **	ns
Grain yield	0.78	ns
HI	ns	−0.66 **
Grain weight	0.60 *	ns
Test weight	0.83 ***	ns
Yellow berry	ns	ns
Average temperature during GF	ns	0.76 ***
N° days with temperature T > 30 °C during GF	ns	0.69 **
Semolina protein percentage	−0.62 **	0.63 **
Semolina dry gluten percentage	ns	0.68 **
Gluten index	ns	−0.68 **
P	ns	ns
W	ns	ns
L	ns	−0.69 **
P/L	ns	ns
Gli:glu	ns	0.61 *
Albumins+globulins	−0.91 ***	ns
Gliadins	ns	ns
Glutenins	0.65 **	ns
Milling yield	0.90 ***	ns

***, significant at *p* ≤ 0.001; **, significant at *p* ≤ 0.01; *, significant at *p* ≤ 0.05; ns, not significant.
